# Interpretations of self-rated health in stroke survivors from a semi-rural community in South East Asia

**DOI:** 10.1080/17482631.2019.1613875

**Published:** 2019-05-23

**Authors:** Kwong Hsia Yap, Narelle Warren, Daniel D. Reidpath, Pascale Allotey

**Affiliations:** a Jeffrey Cheah School of Medicine and Health Sciences, Monash University Malaysia, Bandar Sunway, Malaysia; b School of Social Sciences, Clayton Campus, Monash University, Melbourne, Australia; c South East Asia Community Observatory (SEACO), Monash University, Segamat, Malaysia; d International Institute for Global Health, United Nations University, Kuala Lumpur, Malaysia

**Keywords:** Stroke, cognitive impairment, self-rated health, ethnography, older adults

## Abstract

**Purpose:** Stroke survivors report poorer self-rated health (SRH) compared to the general population but there is limited understanding on what contributes to SRH. This ethnographic study examined the individual and contextual factors that shape stroke survivors’ SRH in a rural middle income country situated in South East Asia. **Methods:** Ethnographic methods which encompasses various data collection methods from different data sources were used in this study to describe the socio-cultural context of 16 stroke survivors living in a rural village. Within this context, the experiences of these participants were then interpreted in terms of what contributed to their perception of health and recovery, juxtaposed with objectively measure physical and cognitive states. **Results**: SRH reflected the post stroke adjustment of stroke survivors. Better SRH was influenced by good post-stroke adjustment that was achieved by a combination of physical functioning, cognitive functioning, emotional well-being and family support. Poorer SRH appear to reflect poor post-stroke adjustment regardless of the objective physical and cognitive states of the stroke survivors. It was also observed that cognitive deficits, though its presence was acknowledged by participants, were usually not taken into account when rating SRH. However, while physical functioning was perceived by participants to directly impact SRH, the presence of cognitive deficits (often in tandem with depressive symptoms) indirectly complicated the recovery of physical functions treasured by participants. **Conclusion:** Stroke survivors reporting poorer SRH warrant further attention and intervention from health practitioners supporting the longer-term needs of stroke survivors in similar settings.

## Introduction

Self-rated health (SRH) is a subjective well-being construct demonstrated to predict mortality (Idler & Benyamini, 1997) and other poorer outcomes, such as dementia (Montlahuc et al., 2011). Usually, single item measures asking people to rate their health in variations of “excellent”, “good”, “fair” or “poor” or even visual analogue scales (VAS) which uses lines in a continuum of scales to denote levels of health status are used to elicit SRH (Bernert et al., 2009; Bowling, 2005; Daniilidou et al., 2010). Due to its simplicity and predictive properties, SRH has been widely used as an overall measure of general health (Jylhä, 2009). As such, SRH was also studied in relation to chronic diseases as a marker of overall health monitoring (Mavaddat, Valderas, van der Linde, Khaw, & Kinmonth, 2014). SRH correlated well with objective health (Hunt et al., 1980), but has been more strongly associated with physical health than mental health (Smith, Avis, & Assmann, 1999). Studies of older adults have shown that mental, social and physical dimensions contributed to an individual’s assessment of their SRH (Mavaddat, Van der Linde, Savva, Brayne, & Mant, 2013), which may involve complex cognitive processes (Jylhä, 2009) that are also influenced by the personal, social and cultural identities of the individual (McMullen & Luborsky, 2006), otherwise known as the environmental and contextual factors that surrounds and shapes the individual. Therefore, SRH may capture elements of health that structured questionnaires may not due to its inclusive nature (Jylhä, 2009). However, SRH when analysed in quantitative study designs were constrained by elements that needed to be predefined (as part of the study design), hampering a better understanding of the issue as other elements that may also contribute to SRH were not be able to be observed as they were not measured. Even so, the ease of administration and its predictive properties for important health outcomes enables SRH to provide insight into the health status of large population studies or in countries with limited resources where detailed measurements of health status may not always be possible.

Stroke recovery is known to be multidimensional and complex process (Vanhook, 2009) and carries long-term impacts on health. Survivors generally rated their health worse, as compared with the stroke-free general population (Larsen, Johnsen, Andersen, & Hjollund, 2016; Mavaddat et al., 2013). While poor physical health contributed significantly to poor self-rated health (Mavaddat et al., 2013), social factors that encourage activities out of the home seemed to moderate the effects of poor physical health (Larsen et al., 2016). Experiential studies have documented how stroke survivors struggled with the impact of bodily physical changes caused by stroke which affected their lives in many ways including their perception of self, their social and even psychological health (Arntzen, Hamran, & Borg, 2015; Carlsson, Möller, & Blomstrand, 2004; Kirkevold, 2002; Nanninga, Meijering, Schönherr, Postema, & Lettinga, 2015). Langhorne and colleagues mapped the effects of stroke using the World Health Organization’s International Classification of Function (WHO ICF) framework, and linked bodily impairments which resulted in limitations and participation in valued activities which then led to lowered perception of overall wellness (Langhorne, Bernhardt, & Kwakkel, 2011). However, bodily impairments and activity limitations did not always predict subjective well-being (Zahuranec, Skolarus, Feng, Freedman, & Burke, 2017). While impairments in bodily functions do contribute to poor participation for some, people with higher levels of impairment have returned to their valued activities (Kubina, Dubouloz, Davis, Kessler, & Egan, 2013), suggesting the influence of environmental and personal factors on participation. Longer term studies have found that stroke survivors living in the community perceived sociability as part of stroke recovery (Arntzen, Borg, & Hamran, 2014). Other studies have focussed on meaningful or valued activities that allow participation of stroke survivors and encourage reintegration into their community, enabling formation of new roles and identities thus rendering feelings of better recovery and well-being (Bouffioulx, Arnould, & Thonnard, 2011; Jellema et al., 2017; Kubina et al., 2013).

Other known but less visible consequences of stroke, such as cognitive deficits, emotional lability, fatigue and other psychosocial symptoms, negatively affected stroke survivors’ perception of self (Carlsson et al., 2004; Carlsson, Möller, & Blomstrand, 2009; Kitzmüller, Häggström, & Asplund, 2013), but were less noticed by research participants perhaps in part due to the effects of stroke itself. Stroke survivors with anosognosia (unawareness of impairments) were more frequently unaware of their cognitive impairments than they were unaware of their motor and sensory impairments (Hartman-Maeir, Soroker, Ring, & Katz, 2002). This may result in the lack of recognition of their cognitive problems and thus this may be left out in considerations regarding their post-stroke recovery (Ellis, Focht, & Grubaugh, 2013). But even in stroke survivors who achieved excellent functional recovery after stroke, post-stroke cognitive impairment is common (Jokinen et al., 2015). Adding to this mix is the tendency for health providers to focus first on motor function recovery, meaning cognitive dysfunction may not be detected (van Dijk & de Leeuw, 2012). The literature on stroke recovery appears to describe the more physical aspects and impact of stroke, partly due to the exclusion of individuals perceived to have moderate to severe cognitive impairment which may affect their abilities to relay information pertaining their experiences. The effects of post-stroke cognitive deficits on SRH and recovery remain vague and seldom examined.

In terms of SRH specifically, the severity of physical deficits in stroke survivors too did not explain fully the perception of poor health (Mavaddat et al., 2018). Studies specifically on stroke survivors and SRH do not take into account much of the personal, social and cultural identities of an individual (as hypothesised by Jylhä (2009) to be contributors of the cognitive processes that forms SRH). Furthermore the commonality across studies of SRH (of stroke survivors) and stroke recovery was that stroke survivors’ perception of recovery and health is dependent on the context and the environment that surrounds them—which can be more inclusively assessed in qualitatively designed studies. To this end, most experiential studies on stroke recovery and SRH have been concerned with communities living in the Western hemisphere, with participants recruited from hospital registries, rehabilitation centres or other health facilities. Studies on the experiences and factors shaping recovery in settings with limited facilities and resources were less explored, resulting in an even more limited understanding of what contributes to good or poor SRH.

The aim of this paper is to explore the individual and contextual factors that shape Malaysian stroke survivors’ self-rated health and their sense of recovery, taking advantage of the versatility of ethnographic methods which enables a rich, in-depth understanding of the phenomena under study. This study provides insights into a setting where there is a lack of information on the experiences of recovery and health after stroke.

## Methods

The data discussed in this article were drawn from a broader ethnographic study on stroke recovery in rural Malaysia, conducted through the South East Asian Community Observatory (SEACO) research platform (Partap et al., 2017), a health and demographic surveillance site located in Peninsula Malaysia. While SEACO collects data on the three main ethnic groups—Malay, Chinese and Indian—the current project focuses on one Chinese community. Stroke recovery is multidimensional and occurs within the socio-cultural context of the stroke survivor. Ethnographic methods were employed to understand the complexities of stroke recovery, and to access populations which may be “hidden” due to post-stroke social impacts. An ethnographic approach which encompasses various data collection methods from various data sources enabled the description of the socio-cultural context that the stroke survivor dwelled in, and within it the interpretation of the experiences of stroke survivors that contributed to their perception of health and recovery.

Stroke survivors were initially identified through an annual health survey conducted by SEACO (Partap et al., 2017), with further stroke survivors recruited through snowball (network) sampling by a community liaison. House-to-house visits were then conducted with potential participants to assess their suitability and willingness to participate in the study.

### Data collection

Data collection had two main components. First, ethnographic data were generated through participant observation of everyday life of older adults living with stroke, the participatory data collection methods of photovoice (Wang & Burris, 1994), and via in-depth interviews (IDIs) with participants, their household members, and other people in their immediate social environments through multiple encounters throughout the data collection period. KHY (who undertook the field research) had lived in the village intermittently during the data collection period and had c`onducted the IDIs within the homes of the participants. Fieldwork was conducted from July 2015 to June 2016 and concentrated in one rural village.

Initially, IDIs focused on participants’ stroke experience (onset, responses, and subsequent actions to their symptoms). Participants’ perceptions of their subjective memory status and recovery were also included in the IDI. The subsequent direction of the IDIs was based on the responses given surrounding their stroke experiences in which relevant issues were explored and probed. For the photovoice component, participants were asked to take images representing their stroke related life experiences, especially objects, places, events and activities which were of importance to them. These images were then used to guide the IDIs and participant observations. Observations on the daily lives of stroke survivors, their actions and reactions towards arising issues and their surroundings were documented through fieldnotes. Multiple interviews were conducted with each participant, ranging from a minimum of two to a maximum of six audio recorded IDIs. Everyday life in the village, as well as interactions of the participating stroke survivors and their immediate physical and social environment were the main focus of the observations and ethnographic enquiry. All observations and fieldnotes were later written up in full.

Second, to capture the current functional state of participants and how this may have influenced how they viewed health and recovery, several scales were also administered and rated. These included:

*Modified Rankin Scale (mRS)* (van Swieten, Koudstaal, Visser, Schouten, & van Gijn, 1988) was used to rate functioning in stroke survivors. The mRS is more highly correlated to mobility and disability than to cognition and social functioning (de Haan, Limburg, Bossuyt, van der Meulen, & Aaronson, 1995). The scale ranges from 0 (no symptoms at all) to 6 (dead). In the current study, participants’ rating scores were between 1, where they experienced symptoms but were recovered enough to perform all previous activities (prior to stroke), to a maximum rating of 4 (moderately severe disability; unable to walk without assistance and unable to attend to own bodily needs without assistance). This study utilized a simplified algorithm developed by Bruno et al. (2010) to rate mRS scores in the participants. Majority of the participants fell into the mRS 2 group, with the median in this group as well. Participants who scored 2 and below were referred to as having better mRS; 3 and above as having poorer mRS (Table I). In this study, mRS was used as the objective measure of physical health.

**Table 1.** Participant characteristics.

Characteristic	N	Health-related factors	N
Sex		Time since first stroke	
Male	10	Less than 1 year	2
Female	6	1 y	4
		2 years	3
		3–5 years	4
		6–10 years	1
		11 or more years	2
Age		mRS score	
50–59 years	1	1–2 (better)	13
60–69 years	5	3–4 (poorer)	3
70–79 years	5		
80–89 years	5		
Marital status		MoCA scores	
Married	13	Below 18 (poorer)	6
Widowed	3	18–30 (better)	8
		Did not complete	2
Household structure		PHQ-9 score	
Lives with spouse only	5	Below 6	12
Lives with spouse and child(ren)	4	6–15	3
and/or grandchild(ren)		16–27	1
Lives in skip generation household (with spouse and grandchild(ren))	4		
Lives with child(ren) and/or grandchild(ren)	3		

*Patient Health Questionnaire, 9 items (PHQ-9)* (Kroenke, Spitzer, & Williams, 2001) was used to probe for symptoms of depression as depression is known to contribute to poor SRH. PHQ-9 incorporates the DSM-IV depression diagnostic criteria with other leading major depressive symptoms (including suicidal ideation) into a brief screen. Participants responded to nine items, with their responses then scored to a maximum obtainable score of 27, with higher scores indicative of more depressive symptoms. In the current study, participants’ scores ranged between 0 and 18, with an average score of 3.3 (see Table I).

*Montreal Cognitive Assessment (MoCA) basic* (Chen et al., 2016; Julayanont et al., 2015) was used as the measure of objective cognition. The MoCA basic was selected because of the extensive adaptation for populations with low literacy that was undertaken by the test creators, in which tasks highly influenced by literacy were taken out or adapted by using more literacy-independent tasks instead. The naming items in the test itself (fruits) were also observed to be fairly relevant and familiar to the older people in this community. The maximum score obtainable through the MoCA is 30, where the higher the score, the better the state of cognition. Previous validation studies of MoCA basic conducted with elderly rural Thai and Chinese populations, cut-offs of 24 and 19 respectively were used to indicate the presence of mild cognitive impairment. As this test had not been validated in Malaysian settings, the resulting scores were not intended to be diagnostic but instead were used to guide to questions regarding participants’ performance in their daily activities with reference to their cognitive abilities. Importantly for this study, the MoCA was used because participants did not receive any clinical assessment during their acute stroke stay in the hospital or in their long-term follow-ups with health service providers. Thus, there were no classifications or indications on the spectrums of cognitive impairment aside from inferences from daily observations. Two participants were unable to complete the screen due to vision impairment. For this article, MoCA assessment scores were divided to better cognition and poorer cognition, the division made at the average point of 18 (among participants in this study). Those who scored 18 and above were referred as having better cognition, and those who scored below 18 were referred to as experiencing poorer cognition.

*EQ5D* (Rabin & de Charro, 2001) *Visual Analog Scale (VAS)* was used to evaluate participants’ self-rated health. Participants were asked to locate their current health state on a scale ranging from 0 to 100%, in which 100% represented their best ever health state and 0% being their worst ever health state. As the average score for the current study participants was 60%, those who rated themselves as 60% and above were considered as being in better health, and those below 60% as being of poorer health.

The ethnographic design of the study which is longitudinal had enabled repeated encounters with participants and their family units; and the triangulation of data collected through different methods in this study served to establish credibility and trustworthiness of the results, as per recommendations (see Lincoln & Guba 1985).

All participants provided written or audio-recorded informed consent. None of the participants underwent any formal cognitive testing to determine cognitive capacity. This has important implications on recruitment and consent process as this study sought to include the voices of people living with cognitive impairment in the study. Consent was obtained in line with recommendations from Beuscher (Beuscher & Grando, 2009) and Hubbard (Hubbard, Downs, & Tester, 2003) on the consenting processes in populations with cognitive impairment. The study was first explained to participants and their immediate family members who cared for them at home. Participants who understand the nature of the study were assumed capable of consent. For participants who were doubted to be able to provide consent, consent was sought from their identified carer and assent was clarified with the participant. This process of consent was negotiated and renegotiated and often took place over multiple encounters throughout the duration of the study, in recognition of the fluctuating nature of capacity (Sherratt, Soteriou, & Evans, 2007) and facilitated by the ethnographic study design. All names of participants and their family members were anonymised and replaced with pseudonyms.

### Data analysis

Data from all sources were integrated and triangulated in the analysis stage (Moran-Ellis et al., 2006; O’Cathain, Murphy, & Nicholl, 2010). Triangulation is a process that allows for comparison and integration of multiple data sources Morse & Field 1995. Participants’ SRH and their objective functional states were tabulated (see Table II). Participants were first grouped into their SRH states of “better SRH” and “poorer SRH”. Within the better and poorer SRH groups, the participants were further compared in terms of their objective health states (cognitive scores using MoCA and physical rating using mRS). Integrated data from the multiple data sources (interviews and fieldnotes) were then compared with SRH status and their objective functional states. Ethnographic data can be analysed via the interpretation and articulation of meaning. This study interpreted meanings through thematic analysis which used measured objective health states as anchors. Themes and subthemes were identified regularities in the data and meanings and were described through the examples in the individual participants by defining variations between “better health” and “poorer health” via the multiple perspectives and combination from the different data sources. Findings were then written up according to themes and subthemes with quotes and excerpts used to illustrate the meanings in a coherent way.

**Table 2.** Participants’ objective health states in relation to self-rated health.

Objectively measured health	Self-rated health	
	Better health (>60%)	Poorer health (≤60%)
mRS		
Good	9	3
Poor	1	3
MoCA		
Good	4	3
Poor	5	2
(2 participants did not complete MoCA)		

## Results

Sixteen stroke survivors, ranging from 3 months to 30 years post-stroke at study initiation, took part in the current study. All were Chinese, comprising of 10 males and 6 females aged from 50 to 84 at the point of first contact. Participants’ characteristics and their responses and ratings from the administered scales were summarized in Table I. Participants’ objective health states in relation to self-rated health were tabulated in Table II.

Participants frequently viewed their health in terms of their physical abilities and capacity, because this was seen as granting them independence in doing the things they were fond of: walking or taking their own transportation to run errands or to socialise, doing house chores, cooking and more. Although they experienced some level of cognitive impairment, participants did not take this into account when discussing their self-rated health. This could be seen when comparing their VAS responses (the indicator for SRH) with MoCA and mRS scores (Table II). Of those who returned a VAS response of 60% and above—who we classified as having “better” SRH—six had poor (less than 18) MoCA scores. In contrast, only one had a poor mRS score, suggesting the privileging of physical functioning in evaluating their self-rated health. What was clear however was that participants who reported “better health” in each of the objectively defined functional categories were able to use their personal and surrounding resources and environment to return to their pre-stroke activities or achieve meaningful post-stroke adjustment. Participants who reported “poorer health” were unable to achieve meaningful post-stroke adjustment as illustrated in the following segments.

### Good physical and cognitive function

#### “Better health” reporters were able to return to all of their pre-stroke activities

The influence of physical functioning on participants’ better self-rated health was reflective of the association between function, personal and social factors. Mr Dian (aged 50 years), for example, had made an almost complete recovery following his stroke three months earlier. He showed little evidence of cognitive impairment (MoCA = 25) or mood changes (PHQ-9 = 0) following stroke. Indeed, Mr Dian had recovered “well”: at the time of stroke, his face drooped and one side of his body became weak; he was also unable to walk without assistance at that time. He had focused on his recovery in the time since then, diligently exercising every morning by walking around the village and swinging his arms. By the time he participated in the study (3 months post-stroke), he had resumed all of his pre-stroke activities, including operating his business, transporting his teenage daughter to and from school, and undertaking his personal errands. High levels of physical recovery facilitated Mr Dian’s social participation. He enjoyed travelling around the village on his motorcycle before opening his store, and was often seen chatting with community members in the village centre in the mornings. In fact, his only reminder of stroke was occasional numbness in his arm. Mr Dian was what one would conventionally expect out of someone who claimed that their health was good.

#### “Poorer health” reporters were unable to return to all of their pre-stroke activities

Another group of participants who drew attention were those who had relatively better mRS and MoCA scores but reported their health as “poorer”. This was the case for three study participants.

##### Unsatisfactory physical recovery

Madam Qin (aged 64) mourned the loss of control she had over her physical abilities:

I am okay actually, but this leg and my arms, they are all against me!! When I try to lift this [her hand], it falls down. I keep telling people, the rest of my body is fine, but this hand and this leg are against me… Now I am suffering… I am suffering because I am unable to walk around well.

Compared to immediately after her stroke, Madam Qin had improved a lot. She lived in the village with her spouse, of similar age. Initially, she had one-sided body paralysis and could not walk on her own, so had to outsource her house-chores as her limb weakness prevented her from doing them. A year after her stroke, she could walk without holding on to her walking stick, and was able to hold cooking utensils to make meals. However, as she tired easily, she had to cut back on heavier cleaning chores. The fear of falling also limited her activities and the extent of physical activities that she would consider undertaking:

I asked people to come and wipe the windows, wash this and that, take the curtains down and I put them into the washing machine to wash. I dare not climb. I need to take care of myself… If die from the fall, [that’s] still okay, but if unable to die, life will be like a dog, don’t you think, like a dog?

She felt that having a fall would result in worse physical weakness than her current state. Even so, she refused to carry a walking stick that could stabilize her during moments of imbalance. She disliked being seen with the walking stick so much that she had limited her social activities out of the house to avoid falling. Her inability to participate in events which were meaningful to her also contributed to a loss of self-worth. As she had considered herself an industrious person who never stopped working and always on the move regardless whether she was in or out of her home, she was bored as she no longer had much to do. Prior to stroke, she would babysit her grandchildren, taking care of their food preparation. She worried that her grandchildren may not get nutritious food as their parents would not be able to prepare home-cooked food for them. Madam Qin derived joy from being able to take care of the needs of her family, and the thought of not being able to perform all of the activities that made her feel valued upset her. She experienced episodes of intense depression where she would voice her suicidal ideations: “Sometimes I feel angry and think… this road (death), one day everyone needs to go through it, it is better I die earlier.” She also became agitated when she found some of the tasks in MoCA difficult:

You should not ask so much, I do not know, later I will get mad… I am telling you frankly… I do not know, these I do not know… you do not ask so much, I do not know.

Her MoCA score (at 18 points) was the average score in this study; however, she may have recognised that her memory was not as good as it used to be, and she seldom talked about it because of the overwhelming loss she felt about her physical abilities. In the corner beside her telephone, the wall was pasted with some telephone numbers of people she would sometimes call. She used to be able to recall those telephone numbers without reminders, but found it more difficult after her stroke. She found ways to compensate for her memory deficits, although she did not like to be reminded of these. However, she was unable to get over the loss of her abilities to do undertake the work she valued (which required more physical force and agility than she could exert) that most affected the her self-rated her health (as poor). Madam Qin’s perception of meaning in life was strongly tied to her contributor as a homemaker and carer for her children and grandchildren.

##### Depression and unsatisfactory physical recovery to pre-stroke state

Physical improvements were not enough in determining the self-rated health of some participants. These participants experienced substantial physical improvements following stroke (Madam Qin included) but were still unable to adjust their expectations to what their current bodies could achieve. Accordingly, they reported depression over the loss of these abilities. Mr Shui (aged 70) scored the highest in the depression scale (PHQ-9 = 18), but also had the second highest MoCA score (at 24 points) and was in the better mRS (rating = 2) group. However, his SRH was in the poorer ratings (VAS = 50%). Although he had recovered from stroke to the extent he could resume his usual activities, and take care of his own business and finances, his body was a lot weaker than before. He no longer carried out full-time work in his farm as he was not able to withstand the exhaustion:

Before my stroke, I was like an ox… [now] I work for 10 minutes, rest for 30 minutes, work for 10 minutes, rest for 30 minutes, like that.

The feeling that he was not completely whole despite assurances from his healthcare provider about his good recovery resulted in lower self-esteem. He walked with a slight limp, and his right eye vision worsened after stroke six years ago. He would sometimes have suicidal thoughts from the loss of self-worth because of these limitations:

I don’t think of anything else, I just think, I should just die… there is no meaning to life.

His wife reported that he would often have mood swings at home and isolate himself from friends and family. This loss of self-worth contributed to his perception of poor self-rated health despite his relatively high functioning.

### Good physical but poor cognitive function

What illuminated the challenges of measurement were the five instances where people scored below average in MoCA but continued to rate their health as “better”, illustrated by three participants in the following paragraphs

#### Poor cognitive scores do not mean poor cognitive functioning in their environment

One participant, Madam Di, a sprightly 81 year old, had lower than average scores in the cognitive screen and was in the better mRS group. She said that her health was “good” (VAS = 80%), but this was not reflected in MoCA (score = 12): importantly, the deficits it captured did not reflect what was important in her life. She felt the physical impacts of stroke, and was unhappy when she was not able to perform things the way she wanted. For example, because of lingering weakness in her hand and leg, she was not able to hold the heavy grass shears or squat and could thus no longer tend to the grass in front of her home. Even though she declared that her memory was fine, she gave a disclaimer when she was not able to perform well in more than half of the cognitive screen, “Sometimes I cannot remember so well, old already”. Other than normalising cognitive deficits as due to ageing, she explained that she was not educated formally, so she just could not get used to the structure of the test. Despite her less-than-optimal performance in the screen, she seemed able to handle other daily activities well enough. She had limb weakness and numbness at stroke onset and now, three months later, was able to walk unaided. She was unsteady at times and admitted to having to hold on to the sides of the walls inside her home. However, she was adamant that she walk outside of her house without any assistance. She huffed at suggestions of bringing a walking stick or umbrella for balance given the slight incline on the road outside her home: “No, I can walk by myself… I can walk by myself… I do not want to bring the umbrella.” She then demonstrated how she made her way out of her home for her walk: she ambulated slowly at first, but grew more confident with each step—thus reinforcing her sense of independence.

While Madam Di treasured the agility that physical health afforded her, some of the activities she valued needed some cognitive precision as well. One day, she happily demonstrated how she cooked lunch, something she no longer did regularly, because her hands were weaker and she was not confident holding a knife. She then detailed how simple it was to prepare a meal of ginger and yam rice, with steamed chicken and soy sauce, and to steam them at the same time. She had even harvested a yam from her vegetable patch for the rice. A fair amount of cutting and chopping were required, and a precise proportioning of the seasonings. She also explained that different variations that can be made with the same ingredients. Her cognitive deficits captured in MoCA did not seem to interfere with her valued activities—cooking and gardening, as described above—while her physical deficits were felt more keenly. This explained, to some extent, her determination and persistence in recovering the deficits she felt more.

#### Family and social scaffolding enabled meaningful functioning

##### Family scaffolding

Madam Lan was in her mid-sixties when she suffered a stroke three years previously. While she had poorer objective cognition scores (MoCA = 13), and was unable to perform most of the recall items from the screen, she had rated her health as “better” (VAS = 80%). However, even at a decade younger than Madam Di, she did not worry about her memory:

Ah, no, not concerned… the person [the individual] is okay, so I am not concerned… When the person does not have any sickness and all that… like this [for her]… I do go and see the doctor… am taking long term medication… so that I do not become demented… I can eat, I can sleep good enough—what more can we ask for? The rest… I just take care of myself, take my bath and feed the old one there [her husband] and sit down and watch the television till night, then I sleep.

For Madam Lan, being self-sufficient was important as her husband (who was suspected to have dementia) was seen as incapable of caring for himself. While she was left with some lower limb weakness after her stroke, she was still mobile within her house, and could move around without help. She was able to care for herself and provide some basic care for her husband such as feeding, changing and bathing her husband (with the help of her son to restrain her occasionally unwilling husband).

While Madam Lan’s MoCA scores were lower than average, she had not experienced the consequences of cognitive deficits because of the lack of complexity in her everyday activities. But cognitive deficits themselves may have led to their seeming insignificance: Madam Lan may not have been able to describe her cognitive deficits because she may not have even noticed the impact of those deficits. This was possibly due to the work undertaken by other members of her household so the cognitive deficits were less noticeable. She lived in a multi-generational home with her son and his family, and was well supported in the household. Her days were spent watching television while the other household members would settle all the other house chores such as cooking, cleaning and purchasing for the household. She also limited her activities to within her home as she was afraid of falling, which she associated with having another stroke. In addition, when she walked, she needed to hold on to walls for stability, as she claimed that her legs wobbled at times and she felt it was always better to have a little support.

Madam Lan’s lack of concern about the physical and cognitive impacts of stroke stemmed from her feeling of being in control of her own affairs. The roles that were not able to be fulfilled by Madam Lan because of her deficits were absorbed seamlessly by the layers of support and “scaffolding” provided by her household members; from taking over strenuous household activities which she no longer had the strength to do to assisting her in the more challenging aspects of care for her husband. In this way, her family members allowed her to maintain a semblance of self and function. Such scaffolding was described by Hydén (2011b, 2011a, 2013) to understand how family members supplemented autobiographical narratives of people with cognitive deficits, to reassert their identities and sense of self. In the same way, Madam Lan’s family provided a supportive environment where she was able to reconstruct a narrative that maintained her sense of identity and meaning in life.

##### Family and community scaffolding

Family structures accommodate and protect the stroke survivor with physical and cognitive deficits in other ways. Mr Ge, who had survived stroke 30 years ago, was left with long-term physical and cognitive deficits. He cheerfully admitted that he had memory problems, and laughed when he was not able to perform some of the tasks in MoCA (below average, score = 16). He rated his health as “good” overall (VAS = 80%): he limped a little, and his thinking was “a little slower” according to his wife, who had gotten used to reminding him on things to be done:

He had a stroke before, so his memory is poorer… it is a bit slower… not like previously… whenever something needs to be done, I will remind him. (Madam Ge)

Life in the village required him to be able to perform tasks needing physical mobility. His left arm and leg had lingering weakness from the stroke, but he had enough strength to complete some tasks like rubber tapping, collecting the rubber milk and transporting the rubber pieces for sale, albeit at a slower rate and with instructions from his wife. The support he received extended beyond the family to a supportive community environment:

When I am a very busy then I will ask him to do things… just tell him what to buy, then he will go and get it… [the shopkeepers] give him more, then we will get some change. (Madam Ge)

The community supported Mr Ge to maintain his independence: because he and his wife had implicit trust in the village stores, in that the shopkeepers were trusted to return the right amount to Mr Ge, she did not worry about Mr Ge getting cheated out of his change and so would give him chores to undertake. He was also in charge of transporting his grandchildren (whom he cared for with his wife) to and fro their school, and managed to keep up with their busy schedule on top of his daily responsibilities. He would send his two school-aged grandchildren (aged 7 and 9) to school each morning, and then collect them home at 1.15pm for lunch. After lunch, he would return the children to school by 2.00 pm, returning for them by 4.15 pm, when he would pick them up and send them to another place within the village by 5.00 pm for another round of tuition. The daily schedule ended with him bringing the children home for dinner around 7.00–8.00 pm.

In contrast to what his below average MoCA scores suggest, Mr Ge was able to take the complexity of their day in his stride. He was usually seen ferrying his grandchildren around on his motorcycle or running errands for his wife. In between his errands, he would chat with other adults also waiting for children outside the school, or share jokes with the traders and shopkeepers while making transactions. He was not able to perform as many tasks as he did before the stroke, but had reconciled himself to his current abilities and showed no signs of longing to return to his pre-stroke state. While his wife provided the necessary scaffolding for him in the form of reminders and instructions, the necessity of caring for his grandchildren ensured he maintained a relatively high physical and social functioning. The village social environment also provided another layer of support. The chronicity of his stroke also played a role in his social functioning: given the slow rate of decline and incremental changes in cognitive deficits, he could adapt.

### Poor physical function (cognition score unknown due to poor eyesight)

#### Chronicity of stroke, attribution to ageing and maintenance of control facilitated “better health”

There was also a participant with worse physical functioning who had rated his health as better. Chronicity affected self-rated health even when physical functioning was poor. Mr Zhao (aged 78 years), who could walk only when aided by his walking frame, had rated his health as “good” (VAS = 80%). He was a chronic stroke survivor, having suffered stroke two decades previously. He refused to complete the cognitive screen, because his eyesight was blurry so he would not be able to see the figures. He admitted that his memory and thinking abilities were not optimal, but explained he could live with it. He had forgotten most of the details of what occurred immediately after stroke, a not-uncommon effect, and was unable able to recall the exact year of his stroke. To him, remembering all these things was not important. He had been paralyzed on one side of his body following his stroke and, according to him, had made all the improvements he could within the first few years post-stroke. He no longer ventured out of his home much.

In describing his health as good, he factored in his age in addition to these dimensions of recovery, where he felt that ageing was related to why his physical abilities were limited, and that his health was as good as he could achieve. Until her death several years before, his wife handled the entire household—meal preparation and all house chores such as laundry, cleaning and grocery shopping—for him. Following her death, his daughter moved in, taking over the chores done by his wife. One thing remained the same, however; he retained control of his own finances from within his home. He scoffed at the suggestion that his daughter acted out of filial duty, as he believed that the monthly stipend that he continued to give her was the real motivator. This sense of control was helpful in maintaining his role as the patriarch and main provider of the family, or as he put it, “the man of the house” and may have explained his sense of his health as good.

#### Unsatisfactory physical recovery hampering pre-stroke activities

Participants who reported poorer health were commonly fixated on physical wellness, For example, Mr Yun (aged 83), nothing was more important than recovering his pre-stroke (2 years prior) physical function. He was left with lower limb weakness and had to use a walking frame to get around, hence his poorer mRS rating (mRS = 3). Blind in one eye and blurry in the other, he could only see the overall outline of people but was unable to make out facial features. At home, his usual activities involved listening to the radio or television as he could no longer read newspapers. He was familiar with the layout of his home and could move around deftly with his walking frame. When asked about his memory, his son, who was seated behind Mr Yun, shook his head (indicating that Mr Yun’s memory was not too good). Mr Yun himself could not really remember the events after his stroke. He was abjectly focused on trying to recover to the extent he could walk without any help: “I do not think about that [my memory problems]. I just want to wait until my leg is better.”

Despite feeling so strongly about his physical loss, he did not identify any of the depressive symptoms in PHQ-9. Pre-stroke, he worked in an elder association where he cleaned tables, served coffee, or did errands for its members (who usually gathered there to play recreational mah-jong). He would also socialise there until it was time for him to go home. He longed to recover so that he could resume this work outside his home, but his fear of falling in an unfamiliar environment kept him from venturing out unless he was accompanied by a family member: “I am afraid of falling, and having another stroke, afraid of that. So I do not go out, I just walk around the house.” To Mr Yun, the only way to be able to resume his activities independently was to restore the strength in his weak leg, so that he could walk stably by himself. Bereft of the opportunity to do things out of his home without assistance, he could only spend his days repeating his routine of exercises and thinking of getting “well”.

### Poorer physical and cognitive functioning

Two participants who reported poorer physical and poorer cognitive functioning had reported their health as “poorer”. While cognitive deficits were not taken into consideration by participants when rating their health, these very deficits impacted their recovery of physical function.

#### Interaction of poor physical and poor cognitive functioning resulting in post-stroke adjustment failure

Madam Lu (aged 80), rated her health as “poorer” (VAS = 20%) and had both poor mRS (rating = 3) and poor MoCA scores (score = 8). She had recovered from her stroke to the extent that she could navigate around with her walking frame, but still had lingering limb weakness and numbness. Her MoCA scores were the lowest in this group of participants, and this was reflected in her daily behaviour. She tended to remember more clearly about her younger days. Her poor memory was noticed by her husband and grandson, but it was attributed to the “deterioration” which routinely happens to older people:

In short, her brain is a little hazy so a bit messy… so she does not know what she is doing*…* her nature has always been like this… and now her memory has deteriorated… her brain nerves, and her whole body has also deteriorated. (Ah Hing, grandson)

These deficits were not a concern because, to them, she had always been this way. She herself did not feel that there was anything amiss with her thoughts. Her husband, Mr Xing (aged 79), thought that her physical state was “good enough”. While it was not what it used to be before stroke (one year ago), he felt that some of her limb numbness and weakness could be mediated by stretching exercises at home but was frustrated that she did not do them on her own. Madam Lu, on the other hand, described her condition as something she had no control over:

Well, you can put it this way … of course I want to do [work] but I am unable to do it, so I have no way. My hand does not… it’s like it’s dumb… so I can’t do it… no strength.

She wanted to “recover more” but she had no idea what she should be doing for better recovery, and despite being told what to do to alleviate symptoms, claimed that these were not effective. She also complained that she did not have anything to do at home, which was why she felt that she was useless: “I cannot do things, now I am old, so it is useless.” Part of this was because her husband took over most of the household chores when she had her stroke.

Madam Lu’s experience demonstrated how scaffolding by family and community was much more ambivalent. Even after she recovered physically well enough to walk around by herself, she was not able to do housework anymore because her family members decided she was still confused and her hands too weak. She could still perform her basic self-care, such as bathing and grooming, but she resented being told what to do and having her opinions overridden by her husband, who felt that some of what she said did not make sense. Yet sociality was important for Madam Lu’s general health: Before she had stroke, she loved to ride her bicycle to neighbours’ homes around the village when she finished her household chores. She was unable to ride her bicycle since her stroke but her friends would visit her at home each week to walk her to (and from) another neighbour’s home for a social visit. While Madam Lu was not always coherent in her conversations, her friends were very inclusive and never criticized her moments of memory lapses. These moments took Madam Lu’s mind off her suffering:

As long as there are people around to talk to her, she is fine. When left alone (at home with him), she goes nuts… complains about pain, wanting to die. (Mr Xing)

Her friends in the village provided a form of support during the moments they spent in conversations, but those moments were brief and forgotten as soon as she was left alone again. While her memory was decidedly poor both objectively and subjectively, her concept of recovery was associated with regaining her physical health, as she associated her valued activities with the nimbleness of her arms and legs. Even though her memory deficits were obvious to her family members, they did not understand how it contributed to her lack of motivation in performing “recovery” activities. In consequence, she was left to self-direct those activities, which of course was impossible. As a result, she was unable to construct a post-stroke identity for herself and was left in a cycle of distress.

## Discussion

This study sought to understand individual and contextual factors that shaped the experience of stroke recovery and self-rated health of older aged stroke survivors. The perception of health and recovery for these participants is complex and multidimensional. Good SRH can be shaped by better physical, cognitive and emotional health but these factors do not explain the perception of good SRH entirely. There were participants with poorer objective cognitive scores and physical functioning (compared to those with poor SRH) who reported good SRH.

Findings were in congruence with the existing literature on SRH (Larsen et al., 2016; Mavaddat et al., 2013, 2018), where functional disabilities alone do not fully explain SRH. This study further extended the findings from a previous qualitative study on the perceptions of SRH in stroke survivors (Mavaddat et al., 2018) in illustrating how the social environment of the family and community facilitated the path to good SRH. The participants with good SRH, despite their functional disabilities managed to reconstruct their self-identity via valued activities which are contextually driven, especially in terms of pre-stroke roles. The family members of these participants played instrumental roles in facilitating these activities seamlessly. Meaningful activities were crucial for the well-being of stroke survivors post hospitalisation (Wood, Connelly, & Maly, 2010) and social support facilitated all types of activities (Jellema et al., 2017), reflecting what was found in this study. For those with good SRH, stroke had become part of the participants’ expected biographical experience—“biographical flow” (Faircloth, Boylstein, Rittman, Young, & Gubrium, 2004)—instead of the biographical disruption (Bury, 1982) experienced by many with chronic illnesses. Those who had rated their health better managed to find continuity and meaning beyond their stroke event. The chronicity of the stroke event also allowed enough time for participants to adjust to new roles. Here, it was found that valued activities were a way for participants to reaffirm their sense of self and their role in life, contributing to better perception of SRH.

Likewise, regardless of their physical and cognitive functioning, the salient theme in participants who perceived to have poor SRH was their sense of biographical disruption, in which pre-stroke activities and abilities were often compared with their post-stroke abilities. Indeed, pre-stroke activities were regarded as the yardstick for recovery by stroke survivors and this contributes to poor post-stroke adjustment (Dowswell et al., 2000). Even participants with good physical and cognitive functioning struggled with depression over their altered state and the longing to return to the state prior to stroke so that they can fulfil the roles that they viewed themselves in prior to stroke. Depression can be an effect of stroke and known to affect sense of well-being in stroke survivors (Zahuranec et al., 2017). Cognitive impairment, while not directly perceived as part of a function that needs recovery, impacts well-being. Cognitive deficits (such as memory and attention) may affect the capability to process and remember the outcome of post-stroke coping responses which keeps the affected individual from forming a new post-stroke identity (Taylor, Todman, & Broomfield, 2011). This would ultimately result in repeated disconfirmation of pre-stroke assumptions and increased distress in the affected individual. Participants with more apparent cognitive deficits also had depressive symptoms; they were unable to compensate with other ways to adapt or adjust their life roles. Some stroke survivors experience anosognosia which affect their self-awareness and capacity to understand their condition, thus reducing their capacity for successful post stroke adjustment. However, some participants with profound cognitive impairment were able to retain a sense of self in their actions and interactions with their surroundings (Kontos, 2012). In Madam Lu’s case, she had difficulties in forming a new post-stroke identity for herself partly due to her cognitive deficits. The sense of self retained by her was in conflict with the physical deficits she sustained after stroke. Her cognitive deficits impaired her thinking and adaptation skills, preventing her from forming a new post-stroke identity. Even though her family was supportive in assisting with the daily care activities, their inability to understand the impact of cognitive deficits on her physical functioning and the depth of her depressive state contributed to her persistent feelings of poor health.

The ethnographic approach taken in this study illustrated the real live context that these participants dwelled in. Interpretations of meanings behind their actions and reactions were enabled in this circumstance, allowing a deeper insight and understanding into the experiences of stroke survivors on how they view recovery.

## Conclusion

In this community, while it is clear that while some stroke survivors succeed in post-stroke adjustment (largely supported by their family and community environment), there were also individuals who still struggle with their post stroke identities even years after the stroke event. Perception of SRH is important as it appears to reflect the post stroke adjustment of stroke survivors, and may be useful for health practitioners in the community in providing support for the post stroke needs of these individuals. In particular, those with poor SRH warrant further attention and intervention as it signified poor post-stroke adjustment of which contributing factors (such as depression and cognitive impairment) should be detected and supported by the health system.
